# Controllable oscillatory lateral coupling in a waveguide-microdisk-resonator system

**DOI:** 10.1038/s41598-017-08656-w

**Published:** 2017-08-14

**Authors:** Fang Bo, Şahin Kaya Özdemir, Faraz Monifi, Jing Zhang, Guoquan Zhang, Jingjun Xu, Lan Yang

**Affiliations:** 1 The MOE Key Laboratory of Weak Light Nonlinear Photonics, TEDA Institute of Applied Physics and School of Physics, Nankai University, Tianjin, 300457 China; 2 Department of Electrical and Systems Engineering, Washington University, St. Louis, Missouri 63130 USA; 3 Department of Automation, Tsinghua University, Beijing, 100084 China; 4 The Collaborative Innovation Center of Extreme Optics, Shanxi University, Taiyuan, Shanxi 030006 China

## Abstract

We report a theoretical and experimental study of coupling between a whispering-gallery-mode (WGM) microdisk resonator and a fiber taper which exchange energies at two distinct regions. We observe an oscillatory behavior in the coupling strength as a function of the distance between the two coupling regions when a fiber taper is moved laterally above the resonator at fixed vertical distance. This oscillation is clearly seen in the linewidth of the resonance as well as in the on-resonance transmission. A theoretical model considering for two-point coupling successfully explains the experimental observations as being a result of the interference between the light fields coupled into and out of the resonator at two distinct regions and the light transmitted through the waveguide. Critical coupling in two-region coupling is a collective result of the coupling at two different coupling regions, and does not require critical coupling at both or at any one of the two coupling regions. This relaxes the conditions for achieving critical coupling in waveguide-resonator systems. The discovery of this previously unnoticed oscillatory behavior in two-region coupling between a WGM resonator and a waveguide will benefit both fundamental studies and practical applications based on WGM resonators.

## Introduction

A resonator coupled to a waveguide constitutes a very simple yet powerful platform to carry out fundamental research on optomechanics^1–3^ and quantum information science^4^ and practical applications ranging from low-threshold lasing^5^ to high-performance sensing^6–10^. Moreover, it is also a basic and highly versatile unit for building more complicated photonic structures and processors, such as add-drop filters^11^, coupled-resonator optical waveguides^12^, and parity-time-symmetric structures for unconventional control of light^13–16^. Besides high quality *Q* factor and small mode volume *V*, maintaining an efficient, stable, and close-to-ideal coupling between the resonator and the waveguide is crucial for the success of the aforementioned studies and applications. Therefore investigating and understanding the behavior of a resonator with a waveguide under various coupling configurations and conditions is of great important from both the fundamental and the practical points of view.

According to the coupling theory proposed by A. Yariv^17^, the normalized transmission *T* of a waveguide-coupled resonator is given by

1 $$T=\frac{|{E}_{{\rm{out}}}{|}^{2}}{|{E}_{{\rm{in}}}{|}^{2}}=\frac{{\alpha }_{0}^{2}-|t{|}^{2}-2{\alpha }_{0}|t|\,\cos \,{\varphi }_{0}}{1+{\alpha }_{0}|t{|}^{2}-2{\alpha }_{0}|t|\,\cos \,{\varphi }_{0}},$$

where *E*_in_ and *E*_out_ are the amplitudes of the light fields in the waveguide right before and after the resonator-waveguide coupling (RWC) region, *α*_0_ = *e* ^−*απr*^ is the inner circulation factor describing the change of the field amplitude after a round-trip in the resonator with *α* representing the equivalent absorption coefficient which includes all the intrinsic losses, such as material absorption and the scattering into the surrounding and the waveguide modes other than the resonant mode (RM) of interest^18^, *r* is the radius of the path the RM travels, *ϕ*_0_ = 2*k*_R_ *πr* is the round trip phase with *k*_R_ = *n*_R_ *k*_0_, *n*_R_ is the effective refractive index of the RM, *k*_0_ = 2*π*/*λ*_0_ indicates the propagation constant in vacuum, *λ*_0_ is the wavelength in vacuum, and *t* is the transmission coefficient through the waveguide and is related to the coupling coefficient *κ* through |*κ*|^2^ + |*t*|^2^ = 1. The coupling coefficient *κ* is mainly determined by the amount of spatial overlap between the RM and the waveguide mode (WG) as well as by how much their propagation constants differ^18–20^.

Note that coupling between a resonator and a waveguide is through their evanescent fields whose strength decreases exponentially with distance. Thus, the RWC becomes stronger when the waveguide approaches the resonator either horizontally or vertically because spatial overlap between the light leaking out from the resonator and that from the fiber taper increases. As the distance between the resonator and the waveguide decreases laterally, the RWC goes from under coupling, where intrinsic loss is higher than coupling loss, to critical coupling point, where intrinsic loss equals to coupling loss, and then to over-coupling regime, where coupling loss is higher^18^. The linewidth of the resonance at the critical coupling is twice the linewidth at the deep undercoupling regime. Recently, M. Ghulinyan *et al*.^21^ showed that the dynamics of the RWC process differs significantly from the conventional picture when the waveguide is moved vertically with respect to the resonator with the lateral distance kept constant. They demonstrated that contrary to the conventional lateral case, the vertical coupling process exhibits oscillatory RWC with alternating under- and over-coupling regions separated by multiple critical points as the vertical distance between the resonator and the waveguide varies. The vertical oscillatory RWC effect was interpreted via the coupling mode theory by considering the RWC as the coupling between two parallel waveguides.

**Figure 1:**
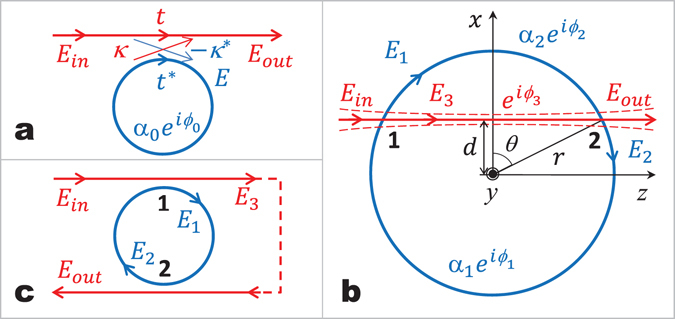
Illustrations of several resonator-waveguide coupling (RWC) systems. (**a**) The simplest RWC system with a single waveguide placed close to a resonator. *t* indicates the transmission coefficient through the waveguide and *κ* chosen to be $i\sqrt{1-{t}^{2}}$ for simplicity is the coupling coefficient. The symbol * denotes the conjugate complex value of *t* and *κ*, respectively. (**b**) Two-point coupling between a single resonator and a single waveguide. *α*_*i*=1,2_ and *ϕ*_*i*=1,2_ are the amplitude and phase changes of the electrical field propagating along different arcs of the resonator, respectively. *ϕ*_3_ represents the phase change of light propagating from point 1 to point 2 through the waveguide. (**c**) The equivalent setup of the two-point coupling system shown in panel b. If the feedback channel indicated by the dashed line is not connected, it is the standard setup of an add-drop filter.

Up to this date discussions concerning the RWC have been limited to two cases: In the first case, the waveguide is placed in the same plane as that of the resonator but outside the resonator’s circular rim and the lateral distance between them is varied^18^. The RWC increases monotonically as the waveguide approaches the resonator before they are in contact. In the second case, however, the resonator is placed in a plane above or underneath the rim of the resonator^21^ and the vertical distance is varied. In both cases, the coupling and the energy exchange between the waveguide and the resonator takes place at only one small region as shown in Fig. 1a.

Here, we investigate a new RWC configuration (Fig. 1b) in which a straight waveguide is placed between the center and the edge of the resonator in a plane parallel to and close to the surface of the resonator. In this case, in contrast to the previous two cases where RWC takes place at one region, the RWC takes place at two different regions marked as 1 and 2 in Fig. 1b. The setup shown in Fig. 1b are similar to nested rings^22^ and compound ring resonators with double couplers^23^, which consist of a resonator coupled to a coplanar curved waveguide via two individual points. The coupling behavior of the resonator influenced by various parameters, such as coupling coefficient at each coupling point, were theoretically investigated. These theoretical investigations have not discussed any effects of shape and position of the waveguide on the coupling, which is essential in practical applications. In this paper, we experimentally studied the two-region coupling behavior between a waveguide and a resonator on different planes and focused more on the untouched aspects, such as the influences of the horizontal position and thickness of the tapered fiber on the coupling, of the previous theoretical studies. We show that one can obtain very efficient RWC as well as critical coupling by placing the waveguide of various thicknesses at different horizontal place even far from the rim of the resonator. The critical coupling in the two-region coupling regime for thicker tapered fibers is due to the collective response of the system at two different coupling regions, and critical coupling at each of the coupling regions is not necessary. As the horizontal position *d* of the waveguide with respect to the center of the resonator is scanned laterally while the vertical height above the resonator plane is kept constant, RWC experiences periodic oscillation in the *d* < *r* region.

## Materials and Methods

### Sample preparation and characterization

The RWC system in our experiments consists of a microdisk whispering-gallery-mode (WGM) resonator and a fiber taper as the coupling waveguide. The microdisk WGM resonator was fabricated on a silica-on-silicon chip following a procedure consisting of a single UV-lithography step and two rounds of HF and XeF _2_ etchings^24^. The thickness of the silica microdisk resonator was 2 *μ*m. The fiber taper was prepared by pulling a single mode optical fiber while it was heated by a hydro-oxygen flame. Optical microscope and scanning electron microscope (SEM) were employed to characterize the topographic features of the microdisk resonator and the fiber taper. A tunable laser in the 1450 nm band was used to probe the resonances and the coupling process. The light at the output port of the fiber was detected by a photodiode. An oscilloscope and a computer were employed to monitor and analyze the transmission spectra. The relative position of the microdisk resonator with respect to the fiber taper was precisely controlled by a 3D piezoelectric stage with a resolution of 100 nm.

### Numerical simulation

A full-vectorial eigenmode solver based on COMSOL software was employed to simulate the resonance frequencies and the distributions of the energy density of the WGMs supported in the microdisk resonator. The structure parameters were set according to the measurements based on the SEM image and optical microscope image of the resonator.

## Results and Discussion

**Figure 2:**
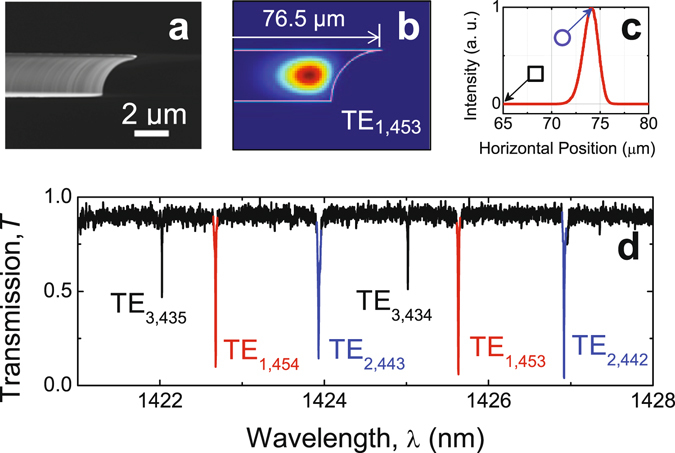
Characteristics of the microdisk resonator. (**a**) The SEM micrograph showing the side view of the edge of the inverted-wedge silica resonator. (**b**) The distribution of the electric energy density of the fundamental mode TE_1,453_. (**c**) The normalized electric energy density of TE_1,453_ just above the top surface of the resonator. (**d**) The measured transmission spectrum showing a few modes.

We first characterized the basic properties of the inverted-wedge microdisk resonator used in the experiments. The radius of the top surface of the inverted-wedge microdisk resonator was measured to be 76.5 *μ*m. The SEM image depicting the side view of the edge of the microdisk WGM resonator is shown in Fig. 2a, which clearly shows the shape of the inverted-wedge resonator providing the structure parameters used in the numerical simulation. A typical transmission spectrum obtained by scanning the wavelength of the input laser is shown in Fig. 2d. The mode numbers were assigned by comparing the measured transmission spectrum with the spectrum calculated using the full-vectorial eigenmode solver. For the sake of simplicity, we concentrated on the coupling behavior of the fundamental mode TE_1,453_. The 2D distribution of the electric field energy density of this mode is given in Fig. 2b. Evanescent wave energy density near the resonator’s top surface is given in Fig. 2c as a function of *x* where we see that in the radial direction the mode distribution of the mode TE_1,453_ has only one intensity maximum at *x* = *r* ~ 74.5 *μ*m.

**Figure 3:**
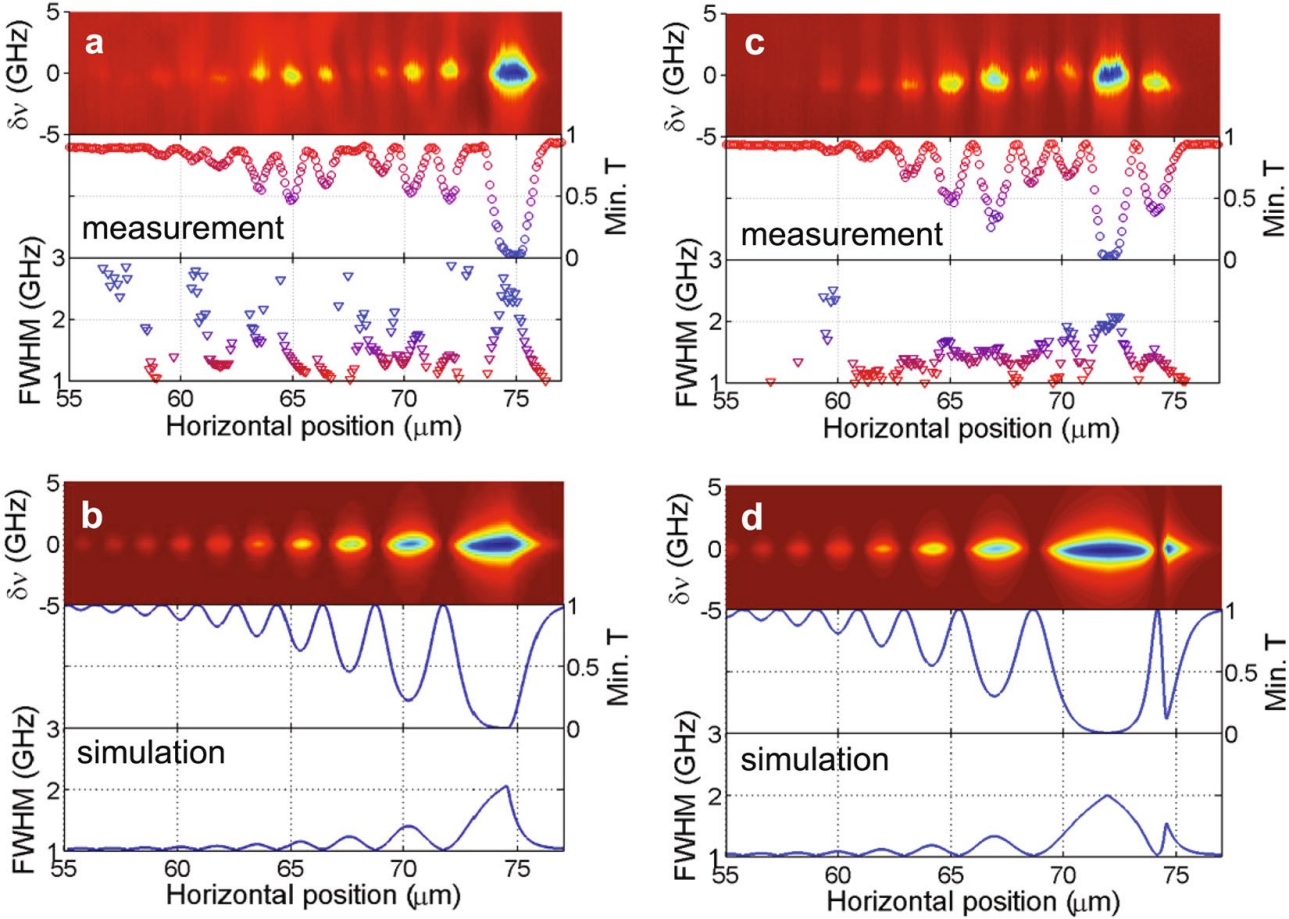
Transmission spectra of a microdisk resonator coupled with fiber tapers of different diameters. (**a** and **c**) Measured transmission spectra and the corresponding minimum transmission *T* and the linewidth (full width at half maximum, FWHM) of the RWC system consisting of a microdisk WGM resonator of radium of 76.5 *μ*m coupled with fiber tapers of diameters of 1.1 *μ*m and 1.2 *μ*m, respectively. FWHM was calculated by fitting the transmission spectra using Lorentzian function. (**b** and **d**) Calculated results of the RWC system consisting of a microdisk with a WGM located at *r* = 74.5 *μ*m and fiber tapers with effective refractive indices equals to *n*_R_ and 1.036*n*_R_, respectively. Other simulation parameters are *n*_R_ = 1.39, *α* = 0.03 cm^−1^, ${\kappa }_{0}=i\sqrt{1-{e}^{-\alpha \pi r\mathrm{/2}}}$, *γ* = *λ*_c_, and central wavelength in vacuum *λ*_c_ = 1425.6 nm.

Using fiber tapers with different diameters we acquired the transmission spectra around the TE_1,453_ mode of the resonator shown in Fig. 2 by laterally moving the fiber tapers in −*x* direction at fixed heights *y* above the resonator surface as shown in Fig. 1b. The center of the resonator was located at *x* = 0, and the lateral move of the tapers was continued until about *x* = 80 *μ*m where the coupling between the taper and the resonator was lost completely. Figure 3a and c present the transmission spectra and the estimated linewidths of the resonances obtained respectively for fiber tapers of diameter 1.1 *μ*m and 1.2 *μ*m, which clearly show the oscillatory behavior of the coupling effect. More importantly, for both of the fiber tapers critical coupling point at which the on-resonance transmission approaches zero was obtained. We observed that as the fiber taper approached the edge of the resonator the spatial overlap between the WGM and the WM monotonically increased and the RWC became stronger. When the fiber taper moved further towards the resonator center, we observed oscillatory RWC manifested with a series of relatively efficient coupling regions separated by some dramatically weak coupling regions. We also noticed that the position, at which the critical coupling point occurred, varied when the diameter of the fiber taper changed. For the thinner fiber taper the critical coupling appeared for *d* ~ 74.5 *μ*m whereas for the thicker one it took place at *d* ~ 72 *μ*m. We attribute this to the fact that the thicker fiber taper has a larger effective refractive index *n*_W_ than the thinner taper so that, compared to the thin fiber, the thicker taper needs to be closer to the center of the resonator to satisfy the matching condition *k*_R_ = *k*_W_ cos *θ* between the wavevector ${\vec{k}}_{R}$ along the tangential direction and ${\vec{k}}_{R}$ along *z* direction when *θ* because the angle *θ* between ${\vec{k}}_{R}$ and ${\vec{k}}_{R}$ increases when *d* decreases. Note that the overlap between the taper mode and the WGM decreases with decreasing *d*. This is similar to the case that the coupling efficiency between two parallel identical straight waveguides are stronger than that between the same two waveguides placed such that they are not parallel to each other^25^. In addition to the difference in the position of the critical coupling, we observe that for *d* > *r* the coupling between the resonator and a thicker tapered fiber is always in the undercoupling regime.

**Figure 4:**
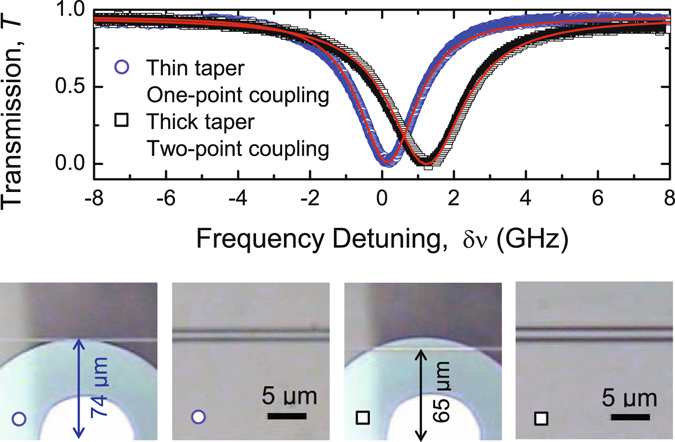
Critical coupling in one-point coupling regime with a thin fiber taper (blue circle) and in two-point coupling regime with a thick fiber taper (black square). The microscope images marked by circle and square, respectively, indicate relative horizontal positions of the fiber tapers with respect to the resonator and the magnified images of the fiber tapers used to achieve critical coupling in the one-point and two-point coupling regimes.

Using a fiber taper of diameter 1.7 *μ*m, we observed efficient energy coupling with on-resonant extinction ratio of −16 dB even when the fiber taper was placed at *x* = 65.0 *μ*m. The results are shown in Fig. 4. As a comparison, the transmission spectrum obtained at the single-point coupling case with a thinner taper of diameter of 1.1 *μ*m placed at *x* = 74.5 *μ*m is also given in Fig. 4. In both cases, on-resonant transmission is very close to zero implying that the system at critical coupling. Enlarged optical images and the horizontal positions *x* of the fiber tapers are shown in the optical micrographs in Fig. 4. As seen in Fig. 2c, the intensity of the TE_1,453_ mode at *x* = 65 *μ*m is negligibly small, thus no efficient light exchange between the microdisk and the fiber taper can take place at this position for the one-point coupling because the WGM and the waveguide modes do not have overlap.

To explain the experimental observations, we developed a theoretical model based on coupled mode theory considering both the one-region and two-region coupling^26^. For simplicity, we assume that the coupling between the resonator and the waveguide takes place at the points 1 and 2 (Fig. 1b). Under the assumption that the coupling is lossless and that a single unidirectional mode of the resonator is excited, the interaction between the WGM resonator and the fiber taper at points 1 and 2 can be described by

2 $$(\begin{array}{c}{E}_{3}\\ {E}_{1}\end{array})=(\begin{array}{cc}{t}_{1} & {\kappa }_{1}\\ -{\kappa }_{1}^{\ast } & {t}_{1}^{\ast }\end{array})(\begin{array}{c}{E}_{{\rm{in}}}\\ {E}_{2}{\alpha }_{1}{e}^{i{\varphi }_{1}}\end{array}),$$

and

3 $$(\begin{array}{c}{E}_{{\rm{out}}}\\ {E}_{2}\end{array})=(\begin{array}{cc}{t}_{2} & {\kappa }_{2}\\ -{\kappa }_{2}^{\ast } & {t}_{2}^{\ast }\end{array})(\begin{array}{c}{E}_{3}{e}^{i{\varphi }_{3}}\\ {E}_{1}{\alpha }_{2}{e}^{i{\varphi }_{2}}\end{array}),$$

respectively. The complex mode amplitudes *E* are normalized, so that their squared magnitudes correspond to the modal power. *α*_1_ = *e* ^−*αr*(*π*−*θ*)^ and *α*_2_ = *e* ^−*αrθ*^ are the inner propagation factor, *ϕ*_1_ = 2*k*_R_ *r*(*π* − *θ*) and *ϕ*_2_ = 2*k*_R_ *rθ* are the phase changes related to the light along the arcs separated by the two coupling points, and *ϕ*_3_ = 2*k*_W_ *r* sin*θ* is the phase shift induced by the light propagation in the waveguide between coupling points 1 and 2. *k*_W_ = *n*_W_ *k*_0_ denotes the light propagation constant in the waveguide and *n*_W_ is the effective refractive index of the waveguide mode. For simplicity, we set *k*_W_ as a constant by neglecting the change of the diameter of the fiber taper with respect to the horizontal position *z*. The loss in the waveguide is neglected as well.

To further simplify the model, all the field amplitudes are normalized to *E*_in_. From Eqs (2) and (3), one can derive the normalized amplitude of the field at the output of the waveguide as

4 $$\frac{{E}_{{\rm{out}}}}{{E}_{{\rm{in}}}}=\frac{{t}_{1}{t}_{2}{e}^{i{\varphi }_{3}}-{\kappa }_{1}^{\ast }{\kappa }_{2}{\alpha }_{2}{e}^{i{\varphi }_{2}}-{\alpha }_{1}{\alpha }_{2}{e}^{i({\varphi }_{1}+{\varphi }_{2}+{\varphi }_{3})}}{1-{t}_{1}^{\ast }{t}_{2}^{\ast }{\alpha }_{1}{\alpha }_{2}{e}^{i({\varphi }_{1}+{\varphi }_{2})}+{\kappa }_{1}{\kappa }_{2}^{\ast }{\alpha }_{1}{e}^{i({\varphi }_{1}+{\varphi }_{3})}}$$

with the transmission given by *T* = |*E*_out_|^2^/|*E*_in_|^2^. Assuming *t*_1_ = *t*_2_ and *ϕ*_1_ + *ϕ*_2_ = 2*mπ*, the condition to obtain the critical coupling is found as

5 $${({t}_{1}^{2}-{\alpha }_{1}{\alpha }_{2})}^{2}+|{\kappa }_{1}^{4}|{\alpha }_{2}^{2}-\mathrm{2(}{t}_{1}^{2}-{\alpha }_{1}{\alpha }_{2})|{\kappa }_{1}^{2}|{\alpha }_{2}\,\cos ({\varphi }_{2}-{\varphi }_{3})=0$$

which is satisfied when *ϕ*_2_ − *ϕ*_3_ = 2*nπ* (n is an integer), *ϕ*_1_ + *ϕ*_2_ = 2*mπ* and ${t}_{1}^{2}-{\alpha }_{1}{\alpha }_{2}=|{\kappa }_{1}^{2}|{\alpha }_{2}$.

Considering the mismatch between ${\vec{k}}_{R}$ and ${\vec{k}}_{W}$, we set the coupling coefficient *κ*_i_ as

6 $${\kappa }_{{\rm{i}}}={\kappa }_{0}{e}^{-\gamma |{k}_{{\rm{W}}}\cos \theta -{k}_{{\rm{R}}}|}$$

in the two-point coupling regime (i.e., *d* < *r* region), where *κ*_0_ represents the strongest coupling one can obtain at either of the two coupling points, ${e}^{-\gamma |{k}_{{\rm{W}}}\cos \theta -{k}_{{\rm{R}}}|}$ indicates that the coupling coefficient *κ*_*i*_ exponentially decays with the increase of the phase mismatch between the tangential component of *k*_W_ at the coupling points and *k*_R_, *γ* is a constant determining the decay rate. When the waveguide is placed at somewhere with *d* > *r*, we assume that RWC is in the one-point coupling regime where the coupling is achieved via a point on the resonator and a point on the waveguide nearest to it, arriving at

7 $$\kappa =2{\kappa }_{0}{e}^{-\gamma |{k}_{{\rm{W}}}-{k}_{{\rm{R}}}|}{e}^{-q(d-r)},$$

where $q={k}_{0}\sqrt{{n}_{{\rm{R}}}^{2}-{n}_{{\rm{S}}}^{2}}$ and *n*_S_ is the refractive index of the surround materials. Equation (7) implies that the coupling coefficient between the resonator and the waveguide exponentially decrease with increasing distance between the waveguide and the resonator.

It is worth mentioning here that the two-point coupling configuration introduced here for a waveguide-coupled resonator is dramatically different than the add-drop filter where two fiber taper waveguides are coupled to the same resonator at different points, forming two one-point coupling and a total of two coupling points. The difference is attributed to the fact that the two-point coupling scheme introduced here is effectively a feedback system, and corresponds to the same situation where the throughput and add ports of an add-drop filter are connected to each other as shown in Fig. 1c. Benefiting from this unique structure, we have demonstrated oscillatory coupling as well as critical coupling achieved with the coupling coefficient of *κ*_i_ at a value that is just sufficient for the resonator to operate in the under-coupling regime for one-point coupling scheme ($|\kappa | < \sqrt{1-{e}^{-2\alpha \pi r}}$) as shown in Fig. 3c and d. Similar phenomenon was reported in theoretical studies on compound ring resonators with double couplers^23^.

Using the the model explained above, we calculated the normalized transmission spectra of the waveguide-coupled resonator with waveguide set at positions with different *d* and with different *k*_W_, which simulates the tapered fiber with different diameters. Figure 3b shows the effect of the position of the fiber on the normalized transmission spectra, the position and the value of the transmission minimum and the resonance linewidth. Note that in these calculations we set *k*_W_ = *k*_R_, implying that the resonator and the waveguide are phase matched. We found that the most efficient coupling was achieved when the taper was put very close to the rim of the WGM resonator as clearly seen in Fig. 3b where the transmission minimum is located at *d* ~ 74.5 *μ*m. Figure 3d presents the results for *k*_W_ = 1.036*k*_R_; the resonator and the waveguide are not phase-matched when the fiber taper is put beyond the rim of the WGM resonator, thus the coupling is weaker than the case with *k*_W_ = *k*_R_. However, when the fiber was moved across the rim towards the center of the resonator, the component of *k*_W_ at the tangential direction of the WGM resonator monotonously reduced, and at a particular position *k*_W_ cos*θ* = *k*_R_, and thus ${e}^{-\gamma |{k}_{{\rm{W}}}\cos \theta -{k}_{{\rm{R}}}|}$ reached 1, where the most efficient coupling efficiency was obtained. This phenomenon is clearly seen in Fig. 3d, where the efficient coupling was realized when fiber taper was placed at *d* ~ 72.0 *μ*m. When the fiber taper was placed above the resonator and *t*_i_ was satisfied, we had the two-point coupling configuration. Horizontal oscillatory coupling effects are also clearly shown in Fig. 3b and d.

It is well known that when the coupling loss and intrinsic loss (sum of all the losses except for the coupling loss) are equal to each other, the resonator-waveguide system is said to be at critical coupling and the transmission becomes zero. It is interesting to note here that in the two-point coupling configuration even though the critical coupling condition is not satisfied at each of the individual coupling points, the system, can still be operated at the critical coupling due to the interference of light fields taking different paths. For example in Fig. 3b and d, the minimum transmission coefficient *t*_i_ at each of the coupling points was equal to *e* ^−*απr*/4^, implying the highest coupling coefficient ${\kappa }_{{\rm{i}}}=i\sqrt{1-{e}^{-\alpha \pi r\mathrm{/2}}}$. In this case, the individual coupling points was far from critical coupling because |*κ*_i_| was smaller than the intrinsic loss of the resonator $\sqrt{1-{e}^{-2\alpha \pi r}}$; however, the combined effect of the coupling points led to observation of the critical coupling for the resonator in two-point coupling regime. Two-point coupling makes a thicker fiber taper, even a side-polished fiber^27^, with higher effective refractive index and lower mode leakage have the possibility to realize efficient coupling with silica resonators.

In Fig. 3, we see some discrepancies between the results from the theoretical model and the experimentally-obtained data, in particular in the period of the oscillatory coupling and the envelope of the transmission minimum as a function of *d*. We attribute these to the fact that the developed model is a very simplified one which does not take into account the change in the spatial overlap of the WGMs and the waveguide modes and other experimental artifacts such as mechanical instabilities and thermal variations which lead to varying coupling efficiencies and resonance wavelength fluctuations as well as the wavelength and intensity fluctuations of the external cavity laser diode used to obtain the transmission spectra. A model which takes those into account will certainly have a better agreement with the experiments.

## Conclusions

In summary, we have introduced a new coupling scheme for the single-waveguide coupled resonator system where a fiber-taper placed at a plane above or blow the resonator can sustain two-point or single-point coupling depending on whether it is placed directly above the resonator or not. The single-point coupling is similar to the traditional approach and exhibits transition from under-coupling to over-coupling regimes through a critical coupling point as the lateral distance between the resonator and the taper is decreased. The two-point coupling, on the other hand, exhibits an oscillatory coupling process and can sustain critical coupling without the requirement that each of the coupling point sustain critical coupling. This is a manifestation of the interference of different paths the light fields takes from the fiber to the detector passing through the resonator. Note that critical coupling is achieved for thicker fiber taper with higher effective refractive index, which gives rise to robust coupling. Our theoretical and experimental results clearly demonstrate the existence of this new coupling behavior which have gone unnoticed up to this date. Because a waveguide-coupled resonator is the basic unit of many more complicated optical processing units and optical sensor networks, we believe that this previously unexplored coupling scheme will help to advance these areas and find usage in applications of microresonators such as tunable feed back system.
